# Right Ventricular Remodeling Using Three-Dimensional Echocardiography in Pediatric Pulmonary Arterial Hypertension Associated with Congenital Heart Disease: A Case–Control Study

**DOI:** 10.3390/jcm15218524

**Published:** 2026-11-02

**Authors:** Carmen Corina Șuteu, Amalia Făgărăşan, Liliana Gozar, Iolanda Muntean, Nicola Şuteu, Daniela Toma, Ana Sorina Paşc, Cristina Blesneac, Laura Beligan, Andreea Cerghit-Paler, Diana Ramona Iurian, Mihaela Iancu

**Affiliations:** 1 Department of Pediatrics III, “George Emil Palade” University of Medicine, Pharmacy, Science, and Technology of Târgu Mureș, 540139 Târgu Mureș, Romania; 2 Department of Pediatric Cardiology, Emergency Institute for Cardiovascular Diseases and Transplantation, 540139 Târgu Mureş, Romania; 3 Department of Medical Informatics and Biostatistics, Iuliu Hațieganu University of Medicine and Pharmacy Cluj-Napoca, 400349 Cluj-Napoca, Romania

**Keywords:** pediatric pulmonary arterial hypertension, right ventricle, three-dimensional echocardiography

## Abstract

**Background:** Three-dimensional echocardiography (3DE) enables comprehensive assessment of right ventricular (RV) remodeling, but its role in children with pulmonary arterial hypertension associated with congenital heart disease (PAH-CHD) remains incompletely characterized. **Methods:** In the current case–control study, eleven children with catheterization-confirmed PAH-CHD and 50 healthy controls underwent conventional, three-dimensional, and speckle-tracking echocardiography. Three-dimensional RV geometry and function were compared between groups, and their discriminative performance and relationships with conventional and deformation indices were evaluated. **Results:** Children with PAH-CHD showed increased RV volumes and transverse dimensions, reduced longitudinal dimension, and impaired systolic function compared with controls (all adjusted *p* < 0.05). RV fractional area change (RV_FAC_3D) and RV ejection fraction (RV_EF_3D) measured by 3DE demonstrated acceptable-to-good discriminative performance (AUC = 0.88, 95% CI: [0.73,1] and AUC = 0.85, 95% CI: [0.70,1], respectively), whereas 3D tricuspid annular plane systolic excursion (RV_TAPSE_3D) and RV_SV_3D did not achieve significant discrimination after correction for multiple comparisons. After controlling for PAH-CHD duration, significant partial Pearson correlations were observed between RV end-systolic volume (RV_ESV_3D) and left ventricular end-diastolic mass (LV_ED_mass_3D): partial r = 0.85, *p* = 0.0017, *p*-corrected = 0.012, LV_ED_mass_3D: RV_SV_3D and RV_Ld_3D were both negatively correlated with LV_AIS (partial r = −0.81, *p* = 0.0042 and partial r = −0.85, *p* = 0.0016, respectively *p*-corrected = 0.0352). **Conclusions:** Most 3D indices discriminated PAH-CHD from controls with acceptable-to-good accuracy. RV volumetric and functional indices correlated broadly with conventional 2D and diastolic Doppler parameters, with LV geometry and segmental mechanics, and, more selectively, with right atrial contractile strain although not all correlations remained significant after correction for multiple comparison. These exploratory findings suggest that 3DE may complement conventional echocardiography in the multiparametric assessment of pediatric PAH-CHD.

## 1. Introduction

Pulmonary arterial hypertension (PAH) in childhood is a rare and progressive disease characterized by pulmonary vascular remodeling and a sustained increase in right ventricular (RV) afterload. Although pulmonary vascular disease initiates the hemodynamic burden, clinical progression and outcome are closely related to the ability of the RV to adapt to chronic pressure overload [1,2,3,4,5]. Accurate characterization of RV remodeling and function is therefore a central component of diagnosis, risk assessment, and longitudinal surveillance in children with PAH [1,3,4,5,6].

Right heart catheterization remains the reference standard for the diagnosis and hemodynamic characterization of PAH, whereas transthoracic echocardiography provides the principal non-invasive modality for serial assessment of cardiac structure and function in children [1,2,3,6,7,8]. Conventional two-dimensional (2D) echocardiographic indices, including tricuspid annular plane systolic excursion (TAPSE), RV fractional area change (FAC), tissue Doppler-derived systolic velocity, and the myocardial performance index (Tei), are widely used to assess RV performance and have demonstrated clinical and prognostic value [2,7,9,10,11]. However, these measurements interrogate only selected components of RV mechanics and are influenced by loading conditions, imaging geometry, and, for some parameters, angle dependence. Consequently, they may not fully characterize the complex contraction pattern of the pressure-loaded RV [6,9,12,13].

Speckle-tracking echocardiography (STE) has expanded the functional assessment of the RV by enabling quantitative evaluation of myocardial deformation [12,13,14]. In pediatric PAH, impaired RV longitudinal strain, particularly RV free-wall strain, has been associated with disease severity, invasive hemodynamics, biomarkers, risk scores, and adverse clinical outcomes [14,15,16,17,18,19,20,21,22,23]. Interpretation in children nevertheless requires consideration of maturational changes in myocardial mechanics and appropriate pediatric reference values [18,19,20,21]. These observations support myocardial deformation imaging as a complementary measure of RV function beyond conventional echocardiographic indices.

The limitations of single-plane assessment are particularly relevant to the RV because its contraction is inherently three-dimensional, integrating longitudinal, radial, and anteroposterior components [12,24,25,26,27,28]. Importantly, three-dimensional echocardiographic studies in healthy children have demonstrated age-related differences in RV contraction mechanics and highlighted the substantial contribution of anteroposterior shortening to global RV ejection [25]. Three-dimensional echocardiography (3DE) therefore provides a conceptually attractive approach to RV assessment by enabling direct volumetric analysis without relying on the geometric assumptions required by conventional 2D methods.

Pediatric reference datasets for 3DE-derived RV volumes and ejection fraction (RV_EF_3D) have increasingly become available [24,27,28]. Moreover, studies in children with PAH have shown that 3DE-derived measures of RV remodeling, systolic function, and deformation can identify abnormal RV mechanics and provide clinically relevant information beyond conventional echocardiographic parameters [29,30,31].

The consequences of PAH are not confined to the RV. Progressive RV pressure overload, chamber remodeling, and interventricular septal displacement may alter left ventricular (LV) filling and mechanics, while right atrial (RA) and left atrial (LA) function may also be affected through changes in ventricular loading and atrioventricular coupling [2,3,7,32,33,34,35]. Studies evaluating ventricular and atrial deformation suggest that these abnormalities provide complementary information regarding the cardiovascular response to elevated pulmonary vascular load [16,17,32,33,34,35,36]. An integrated assessment of RV geometry and function together with biventricular and atrial mechanics may therefore provide a more complete characterization of the cardiac phenotype in pediatric PAH.

Although previous pediatric studies have demonstrated the utility of 3DE for assessing RV volumes, systolic function, and myocardial mechanics in pulmonary hypertension, the integration of 3D RV geometry and function with conventional echocardiographic indices and biventricular and atrial deformation remains less extensively investigated in children with PAH associated with congenital heart disease (PAH-CHD) [29,30,31]. In particular, the relationships among these complementary measures have not been fully characterized in this population. We therefore aimed to comprehensively assess RV remodeling and systolic function using 3DE, alongside conventional echocardiography and myocardial deformation imaging, and to explore their interrelationships and the ability of 3DE-derived RV indices to distinguish children with PAH-CHD from healthy controls.

## 2. Materials and Methods

### 2.1. Study Design and Population

This prospective, single-center case–control study was conducted at the Department of Pediatric Cardiology, Emergency Institute for Cardiovascular Diseases and Transplantation, Târgu Mureș, Romania, a national tertiary referral center for pediatric pulmonary hypertension, between January 2025 and January 2026. The study was designed to characterize RV geometry and function using 3DE in children with PAH-CHD, complemented by conventional echocardiography and myocardial deformation imaging.

The study population comprised 11 children with PAH-CHD and 50 healthy controls. Among patients with PAH-CHD, seven had Eisenmenger syndrome and four had persistent or recurrent PAH following surgical repair of the underlying CHD. Congenital heart defects included ventricular septal defect (VSD), atrioventricular septal defect (AVD), atrial septal defect (ASD), patent ductus arteriosus (PDA), and transposition of the great arteries (TGA), either as isolated lesions or in combination.

Children younger than 18 years with an established diagnosis of PAH-CHD confirmed by right heart catheterization according to contemporary diagnostic criteria were prospectively enrolled [1,2,3,7]. Eligibility additionally required a complete echocardiographic examination of sufficient image quality for conventional, three-dimensional (3D), and speckle-tracking analyses. Patients were excluded when relevant concomitant cardiovascular or systemic conditions other than the underlying CHD could independently and substantially affect ventricular function, or when image quality was insufficient for reliable 3DE reconstruction or myocardial deformation analysis.

The control group comprised 50 children without structural heart disease who underwent cardiac evaluation for an innocent murmur, non-cardiac chest pain, palpitations, or preparticipation sports screening. Normal cardiac anatomy and function were confirmed by comprehensive clinical and echocardiographic assessment. All children with PAH-CHD evaluated during the study period were consecutively enrolled, with no exclusions due to insufficient image quality. All 61 participants (11 children with PAH-CHD and 50 healthy controls) had complete, analyzable datasets for conventional echocardiography, 3DE, and speckle-tracking analyses, including biventricular and atrial strain assessment. No datasets were excluded from the final analyses.

The study was conducted in accordance with the Declaration of Helsinki and was approved by the Ethics Committee of the George Emil Palade University of Medicine, Pharmacy, Science, and Technology of Târgu Mureș (No 3589). Written informed consent was obtained from the parents or legal guardians of all participants before enrollment.

### 2.2. Clinical Assessment

All participants underwent a standardized clinical assessment at study enrollment. Demographic and anthropometric data included age, sex, weight, height, body surface area (BSA), and body mass index (BMI). Resting heart rate (HR), systolic and diastolic blood pressure (sBP, dBP), and peripheral oxygen saturation (SaO_2_) were recorded as part of the clinical evaluation.

For children with PAH-CHD, additional disease-specific data included age at diagnosis, duration of PAH, underlying congenital heart defect, PAH-CHD subtype, clinical symptoms, World Health Organization functional class (WHO-FC), and PAH-targeted therapy.

Functional capacity was assessed using the six-minute walk test (6MWT), with walking distance, SaO_2_, and HR recorded according to the study protocol.

### 2.3. Echocardiographic Assessment

All participants underwent comprehensive transthoracic echocardiography using a Philips EPIQ CVx ultrasound system equipped with an X5–1 transducer (Philips Healthcare, Andover, MA, USA). Conventional 2D, Doppler, 3D, and STE datasets were acquired during the same examination and subsequently analysed using dedicated QLAB software (version 15.0; material number 453562090801). All examinations and measurements were performed by the same experienced pediatric cardiologist in accordance with current ASE and EACVI/AEPC recommendations [37,38]. Acquisitions were ECG-gated when required by the imaging modality and optimized for image quality and temporal resolution. Image acquisition and analysis were optimized to ensure adequate myocardial and endocardial delineation, minimize respiratory and stitching artifacts, and achieve reliable tracking quality for quantitative assessment.

#### 2.3.1. Conventional Two-Dimensional and Doppler Echocardiography

Conventional 2D, pulsed-wave Doppler, and tissue Doppler echocardiography was performed according to current pediatric echocardiographic recommendations [37,38].

RV systolic function was assessed using two-dimensional tricuspid annular plane systolic excursion (TAPSE_2D), RV fractional area change (RV_FAC_2D), the RV myocardial performance index (RV_Tei_2D), and tricuspid annular peak systolic tissue Doppler velocity (Tr_S). RV_Tei_2D was calculated from pulsed-wave Doppler recordings as the sum of isovolumic contraction and relaxation times divided by ejection time. RV diastolic function was evaluated using early and late tricuspid inflow velocities and their ratio (Tr_E/A), tissue Doppler-derived early and late diastolic annular velocities (Tr_E′ and Tr_A′), the Tr_E/E′ ratio, and the isovolumic contraction and relaxation times (Tr_IVCT and Tr_IVRT).

LV systolic function was assessed using mitral annular plane systolic excursion (MAPSE) and mitral annular peak systolic tissue Doppler velocity (Mi_S). LV diastolic function was evaluated using early and late mitral inflow velocities and their ratio (Mi_E/A), tissue Doppler-derived early and late diastolic annular velocities (Mi_E′ and Mi_A′), the Mi_E/E′ ratio, and the corresponding isovolumic contraction and relaxation times (Mi_IVCT and Mi_IVRT).

Measurements were obtained over three consecutive cardiac cycles and averaged for analysis.

#### 2.3.2. Three-Dimensional Echocardiographic Assessment of Right and Left Ventricular Geometry and Function

Three-dimensional RV full-volume datasets were acquired from an RV-focused apical four-chamber view (A4C), with particular attention to complete inclusion of the RV cavity throughout the cardiac cycle. ECG-gated four-beat full-volume acquisitions were obtained during end-expiration whenever feasible to minimize respiratory motion and stitching artifacts. Datasets with incomplete RV visualization or significant acquisition artifacts were excluded from quantitative analysis. Following acquisition, RV datasets were analysed offline using dedicated QLAB software (version 15.0; material number 453562090801, Philips Healthcare). After initialization using standard anatomical landmarks, automated endocardial contours were generated and visually inspected throughout the cardiac cycle. Manual corrections were performed when necessary to ensure accurate endocardial tracking.

**Figure 1:**
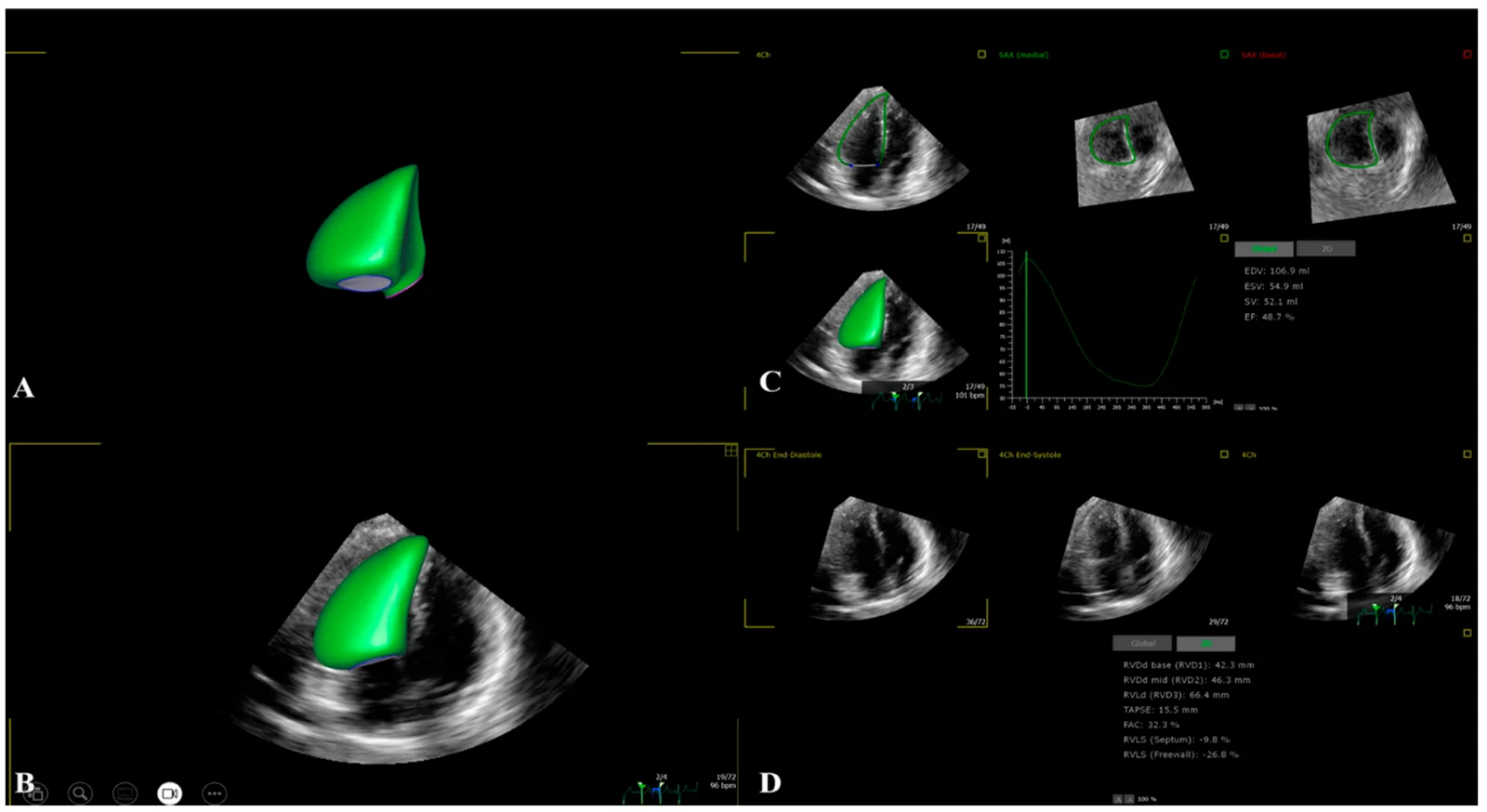
Three-dimensional echocardiographic assessment of right ventricular volumes and function. Representative three-dimensional echocardiographic analysis of the right ventricle (RV). (**A**) Three-dimensional reconstruction of the RV endocardial surface, en-face view. (**B**) The same reconstruction superimposed on the two-dimensional RV-focused apical four-chamber acquisition. (**C**) Three-dimensional quantification of RV volumes and systolic function, including end-diastolic volume (EDV), end-systolic volume (ESV), stroke volume (SV), and ejection fraction (EF). (**D**) Three-dimensional assessment of RV linear dimensions and systolic performance, including basal (RVDd base (RVD1)), mid-cavity (RVDd mid (RVD2)), and longitudinal (RVLd (RVD3)) diameters, tricuspid annular plane systolic excursion (TAPSE), fractional area change (FAC), and septal and free-wall longitudinal strain (RVLS (Septum), RVLS (Freewall)).

The reconstructed 3D RV model provided end-diastolic volume (RV_EDV_3D), end-systolic volume (RV_ESV_3D), stroke volume (RV_SV_3D), and ejection fraction (RV_EF_3D). RV geometry was further characterized by the end-diastolic basal diameter (RV_Dd_base_3D), mid-cavity diameter (RV_Dd_mid_3D), and longitudinal dimension (RV_Ld_3D). Additional indices of RV systolic function, including fractional area change (RV_FAC_3D) and tricuspid annular plane systolic excursion (RV_TAPSE_3D), were automatically generated by QLAB software as part of the 3D RV reconstruction analysis (Figure 1).

**Figure 2:**
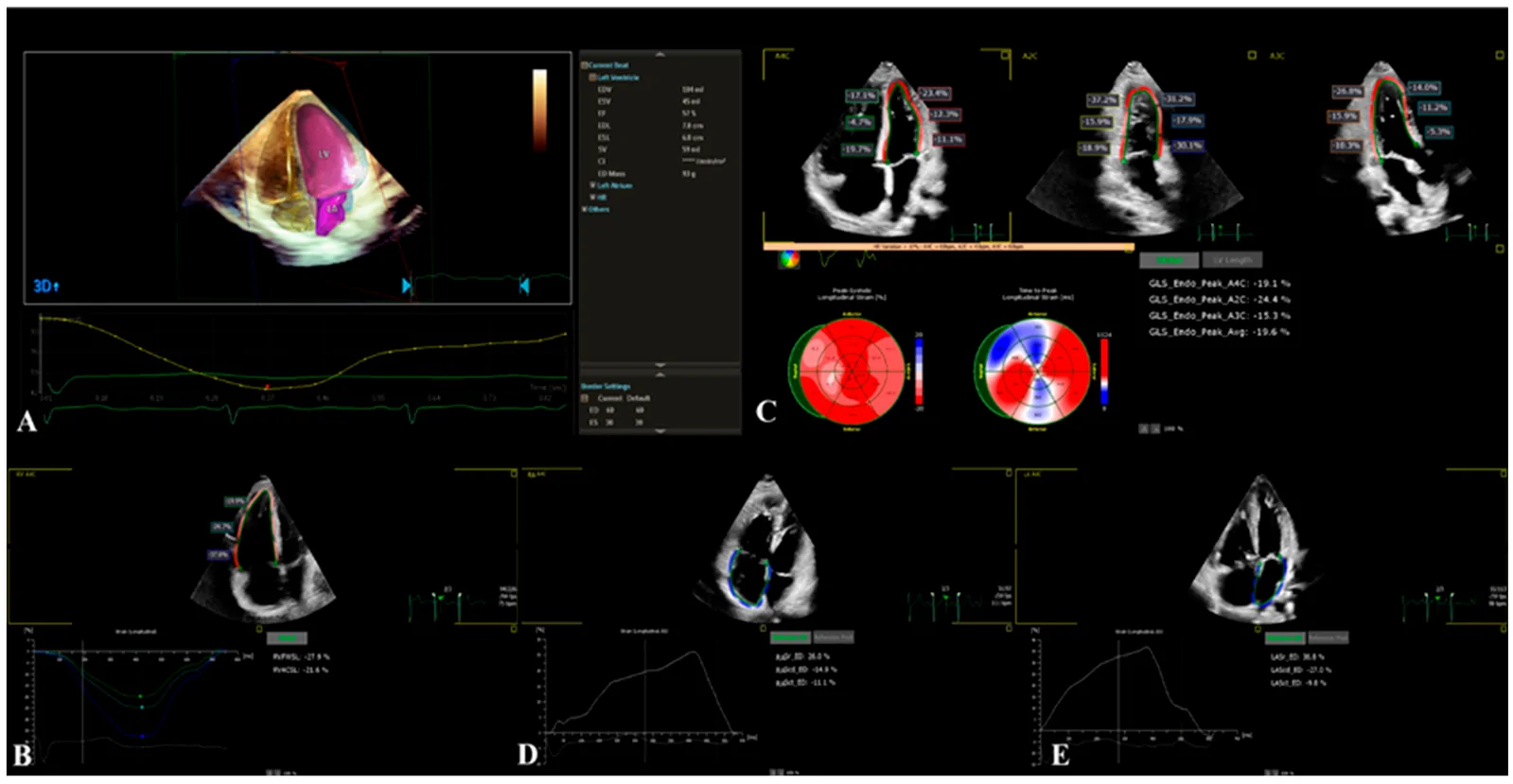
Three-dimensional assessment of left ventricular geometry and function, and speckle-tracking echocardiographic analysis of biventricular and biatrial myocardial deformation. Representative three-dimensional echocardiographic analysis of the left ventricle (LV) and speckle-tracking echocardiographic analysis. (**A**) Three-dimensional reconstruction of the left ventricle (LV) with quantification of LV volumes (EDV, ESV), dimensions (EDL, ESL), mass (ED Mass), stroke volume (SV) and ejection fraction (EF) across the cardiac cycle. (**B**) Speckle-tracking analysis of right ventricular free-wall longitudinal strain (RVFWSL) and right ventricle four-chamber longitudinal strain (RV4CSL) from the apical four-chamber view. (**C**) Speckle-tracking analysis of LV global longitudinal strain (GLS_Endo_Peak_Avg) from the apical four-(GLS_Endo_Peak_A4C), two-(GLS_Endo_Peak_A2C), and three-chamber (GLS_Endo_Peak_A3C) views, with corresponding bull’s-eye polar maps of regional peak systolic strain. (**D**) Speckle-tracking analysis of right atrial strain, including reservoir (RASr_ED), conduit (RAScd_ED), and contractile (RASct_ED) components. (**E**) Speckle-tracking analysis of left atrial strain, including reservoir (LASr_ED), conduit (LAScd_ED) and contractile (LASct_ED) components; ED: end-diastole.

Three-dimensional LV datasets were acquired separately from an apical window using ECG-gated three-beat full-volume acquisitions. LV datasets were analysed offline using the same QLAB software, with automated endocardial contours visually inspected throughout the cardiac cycle and manually adjusted when necessary. The resulting measurements included LV end-diastolic and end-systolic volumes (LV_EDV_3D and LV_ESV_3D), stroke volume (LV_SV_3D), ejection fraction (LV_EF_3D), end-diastolic and end-systolic longitudinal dimensions (LV_EDL_3D and LV_ESL_3D), and end-diastolic LV mass (LV_ED_mass_3D) (Figure 2A).

All 3D datasets were acquired and analysed by the same investigator using the same ultrasound platform and standardized acquisition and analysis procedures.

#### 2.3.3. Speckle-Tracking Echocardiographic Analysis

Two-dimensional STE was performed using dedicated AutoStrain software QLAB 15. Grayscale cine loops were acquired at frame rates > 60 frames/s, with at least three consecutive cardiac cycles stored for each apical view. Automated myocardial contours were visually inspected, and manual adjustments were performed when necessary to ensure adequate tracking.

RV longitudinal deformation was assessed from an RV-focused A4C. Segmental longitudinal strain was obtained at the basal (BRV), mid-ventricular (MRV), and apical (ARV) levels. RV free-wall longitudinal strain (RVFWSL) and RV four-chamber longitudinal strain (RV4CSL) were also recorded (Figure 2B).

LV longitudinal deformation was assessed from A4C, two-chamber (A2C), and three-chamber (A3C) views using the standard 17-segment model. Segmental longitudinal strain was obtained for the basal, mid, and apical levels of the inferoseptal, anterolateral, inferior, anterior, inferolateral, and anteroseptal walls, as appropriate for each apical view. View-specific longitudinal strain and LV global longitudinal strain (LV_GLS) were subsequently derived (Figure 2C).

RA and LA deformation were assessed from the A4C view using 2D STE. Reservoir (Sr), conduit (Scd), and contractile (Sct) strain components were evaluated using both end-diastolic (ED) and atrial-contraction (AC) reference points, yielding RASr_ED, RAScd_ED, RASct_ED, RASr_AC, RAScd_AC, and RASct_AC for the RA, and the corresponding LASr, LAScd, and LASct parameters for the LA (Figure 2D,E). Because a dedicated RA analysis preset was unavailable, RA deformation was quantified using the vendor’s LA analysis template, with the resulting parameters relabeled according to RA nomenclature. This approach was applied consistently across participants, although the software was not specifically designed for RA geometry.

All speckle-tracking analyses were performed by the same investigator using a standardized acquisition and analysis protocol.

### 2.4. Study Objectives

The primary objectives were (1) to compare echocardiographic 3D RV functional indices (RV_EDV_3D, RV_ESV_3D, RV_EF_3D, RV_SV_3D, RV_TAPSE_3D, RV_FAC_3D, RV_Dd_base_3D, RV_Dd_mid_3D, RV_Ld_3D) between children with PAH-CHD and controls, adjusted for BSA, and (2) to assess the discriminative ability of 3D RV functional indices for differentiating children with PAH-CHD from controls.

The secondary objectives were (3) to evaluate the correlation between 3D RV indices and conventional 2D RV parameters and RV strain indices in children with PAH-CHD; (4) to test the correlation between 3D RV indices and conventional 2D LV parameters, 3D LV indices, and LV strain indices in children with PAH-CHD; and (5) to assess the correlation between 3D RV indices and LA and RA strain indices in children with PAH-CHD.

### 2.5. Statistical Analysis

The distributions type of the 3D RV indices, conventional 2D RV parameters and RV strain indices was assessed using the descriptive statistics, quantile–quantile plots (Q-Q plots) and Shapiro–Wilk’s test in each studied group. Variables that followed a normal distribution were reported using the arithmetic mean and standard deviation (SD) while variables that deviated from a Gaussian distribution were summarized as median (interquartile range, IQR: [Q1, Q3]). Qualitative characteristics were described using absolute and relative frequencies (%).

Student’s *t*-test or Wilcoxon rank sum test with continuity correction on independent samples were used to compare demographic, anthropometric and clinical characteristics between controls and PAH-CHD group. Differences in frequency distributions of clinical characteristics between control and PAH-CHD groups were tested using Chi-square or Fisher’s exact test. Right ventricular end-diastolic, end-systolic and stroke volumes were indexed to body surface area (BSA) and expressed in mL/m^2^. Indexed values of 3D volumetric parameters were compared using Student-*t* test for independent samples. Comparisons of 3D RV indices, conventional 2D RV parameters and RV strain indices between groups were performed using analysis of covariance (ANCOVA, type III sum of squares) having group as fixed factor and BSA z-score as covariate. Correlations between echocardiographic measures were assessed using partial Pearson or Spearman correlation coefficients, adjusting for disease duration in PAH-CHD group.

The discriminative ability of echocardiographic indices for differentiating between PAH-CHD children from controls was evaluated using Receiver Operating Characteristic (ROC) curve analysis. The area under the curve (AUC) and its 95% confidence interval were estimated using the DeLong method and the optimal cutoff value was calculated using the Youden index. Pairwise comparisons between ROC curves were performed using DeLong test. The 95% confidence intervals for the corresponding sensitivity and specificity were calculated using bootstrap resampling or Clopper–Pearson method.

All descriptive and inferential statistical analysis were performed using R software version 4.6.1. (R Foundation for Statistical Computing, Vienna, Austria).

All two-sided statistical tests used a significance level (α) set at 0.05.

For all analysis involving multiple comparisons, *p*-values were corrected using the Benjamini–Hochberg method. For the exploratory correlation analysis, the Benjamini–Hochberg (BH) procedure was applied separately within families of comparisons defined by each 3D RV parameter and each category of echocardiographic measures (conventional echocardiographic metrics, LA and RA strain parameters, conventional 2D echocardiographic parameters). A significant result was achieved when estimated significance level *p*-value < 0.05; when the BH procedure was applied, significance was based on the corrected *p*-value (*p*-corrected < 0.05).

## 3. Results

A group of 11 pediatric patients with PAH-CHD were enrolled in the current study, including 5 boys and 6 girls (Table 1). The age values in the PAH-CHD group ranged from 8 years to 15 years at study entry with a mean value (SD) of 11.73 (2.15) years. Patients were diagnosed at a mean age (SD) of 7.91 (4.06) years resulting in a mean disease duration (SD) of 3.82 (3.40) years. A total of 50 healthy children were enrolled in the study with range of age between 4 years and 17 years at study enrollment with an average age (SD) of 10.7 (3.59) years. The distribution of age, sex, BSA and BMI did not differ significantly between the two studied groups (Table 1). Invasive hemodynamic measurements obtained by heart catheterization are presented in Supplementary Table S1. In the PAH-CHD group the mean pulmonary arterial pressure was 48.09 ± 8.15 mmHg and the pulmonary vascular resistance index was 12.06 ± 4.06 WUm^2^.

After adjustment for BSA z-score, children with PAH-CHD had significantly higher means of RV_EDV_3D, RV_ESV_3D, RV_SV_3D, RV_Dd_base_3D, RV_Dd_mid_3D RV functional indices compared with control group (*p* < 0.05, Table 2).

### 3.1. Discriminative Ability of 3D RV Functional Indices to Differentiate Children with PAH-CHD from Controls

**Figure 3:**
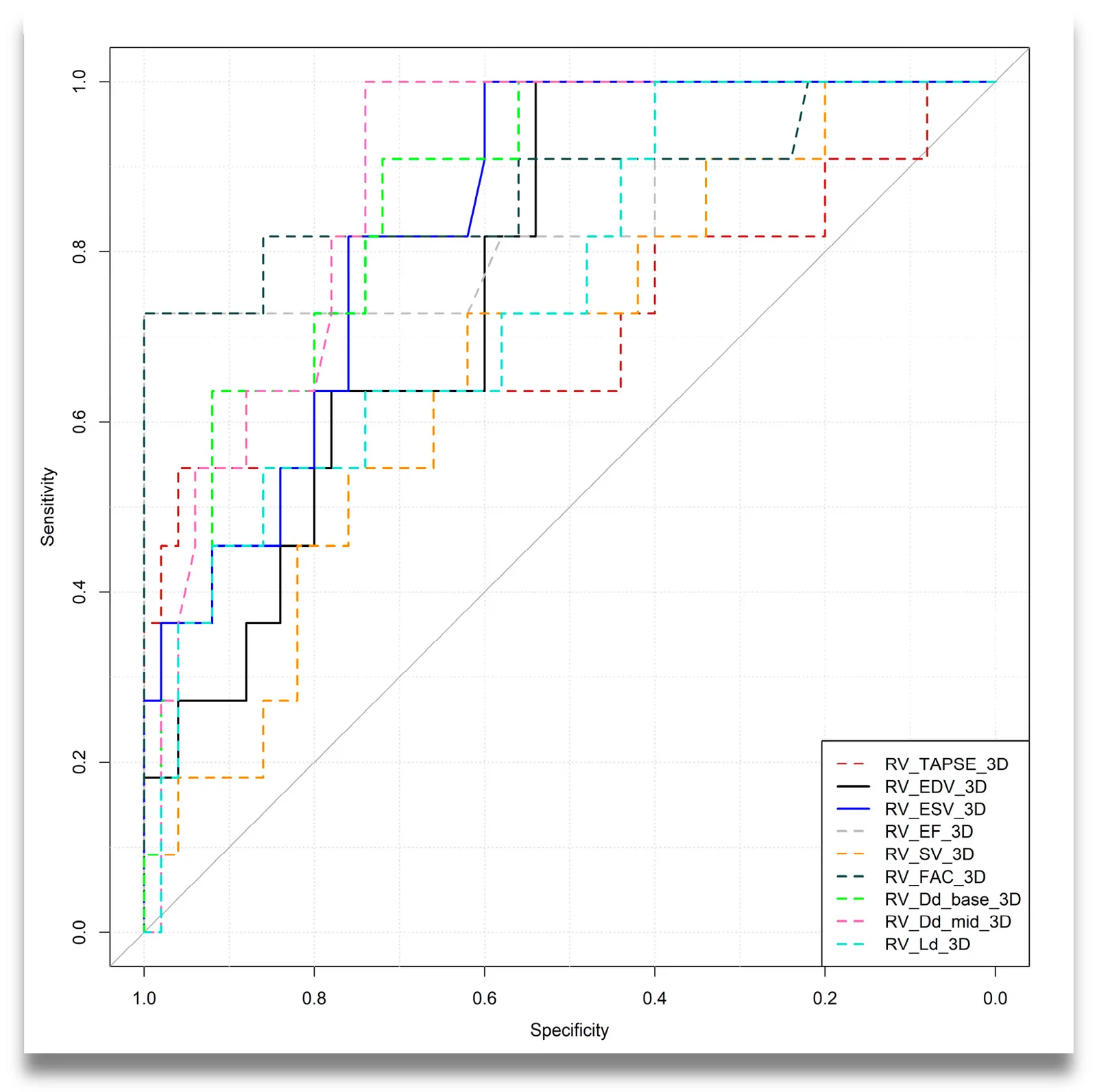
Receiver operating characteristics (ROC) curves of right ventricular functional indices obtained by 3D echocardiography. Note: Curves are color-coded by index (RV_TAPSE_3D, RV_EDV_3D, RV_ESV_3D, RV_EF_3D, RV_SV_3D, RV_FAC_3D, RV_Dd_base_3D, RV_Dd_mid_3D, RV_Ld_3D); the diagonal gray line represents the line of no discrimination (AUC = 0.5). The *x*-axis is plotted in decreasing order of specificity (1.0 to 0.0), following standard ROC-curve convention. Corresponding AUC values, optimal cut-offs, and sensitivity/specificity estimates are reported in Table 3. RV_TAPSE_3D: tricuspid annular plane systolic excursion obtained by 3D echocardiography; RV_EDV_3D: right ventricle end-diastolic volume measured by 3D echocardiography; RV_ESV_3D: right ventricle end-systolic volume measured by 3D echocardiography; RV_EF_3D: right ventricle ejection fraction measured by 3D echocardiography; RV_SV_3D: right ventricle stroke volume measured by 3D echocardiography; RV_FAC_3D: right ventricle fractional area change obtained by 3D echocardiography; RV_Dd_base_3D: right ventricle basal diameter measured at the basal third of the right ventricle in end-diastole by 3D echocardiography; RV_Dd_mid_3D: right ventricle mid-cavity diameter measured at the mid-ventricular level of the right ventricle in end-diastole by 3D echocardiography; RV_Ld_3D: right ventricle longitudinal distance from the tricuspid annulus to the right ventricle apex in end-diastole measured by 3D echocardiography.

Except for RV_TAPSE_3D and RV_SV_3D, all 3D RV functional indices demonstrated a significant discriminative ability differentiating children with PAH-CHD from controls (Table 3, Figure 3). Pairwise comparisons using DeLong test identified a significant difference between the ROC curves for RV_EDV_3D and RV_ESV_3D (DeLong test, *p* = 0.0452). However, after correction for multiple comparisons, no significant difference was found (*p*-corrected > 0.05).

### 3.2. Correlations Between 3D RV Indices and Conventional 2D RV Parameters and RV Strain Indices in PAH-CHD Group Adjusted for Disease Duration

After controlling for PAH-CHD duration, RV_FAC_2D was significantly positively linearly correlated with RV_EF_3D (partial r = 0.88, *p* = 0.0007, *p*-corrected = 0.007). Tricuspid annular early diastolic velocity (E′wave) obtained by tissue Doppler echocardiography (Tr_E′) showed a significant negative partial linear correlation with RV_EDV_3D, RV_ESV_3D, RV_SV_3D, RV_Dd_base_3D and RV_Ld_3D (Table 4). However, after Benjamini–Hochberg correction, only the linear correlation between Tr_E′ and RV_Ld_3D remained statistically significant (*p*-corrected = 0.042). The ratio between early tricuspid inflow velocity and tricuspid annular early diastolic velocity (E/E′) showed a significant positive partial linear correlation with RV_EDV_3D, RV_ESV_3D, RV_Dd_base_3D and RV_Dd_mid_3D. However, after Benjamini–Hochberg correction, only the correlation with RV_Ld_3D remained statistically significant (*p*-corrected = 0.042). Regarding strain parameters, MRV showed a significant positive partial linear correlation with RV_Dd_mid_3D (partial r = 0.67, *p* = 0.0333), although the correlation did not remain statistically significant after correction for multiple comparisons (*p*-corrected *p* = 0.1332).

### 3.3. Correlation Between 3D RV Indices and LV Conventional 2D Echocardiographic Parameters, 3D LV Echocardiographic Parameters and LV Strain Indices

Right ventricle end-diastolic volume obtained by 3D echocardiography (RV_EDV_3D) was significantly positively linearly correlated with MAPSE (partial r = 0.66, *p* = 0.0384) but the correlation did not remain significant after BH procedure (*p*-corrected = 0.3072), and LV_ED_mass_3D (partial r = 0.89, *p* = 0.0006, *p*-corrected = 0.0042). Significant positive partial Pearson correlations were observed between RV_ESV_3D and LV_ED_mass_3D (partial r = 0.85, *p* = 0.0017, *p*-corrected = 0.012), RV_SV_3D and LV_EDV_3D (partial r = 0.73, *p* = 0.0165, *p*-corrected = 0.0824), RV_TAPSE_3D was significantly negatively correlated with LV apical inferoseptal segment longitudinal strain but the correlation did not remain significant after BH procedure (LV_AIS, *p* = 0.0227, *p*-corrected = 0.4994) after controlling for PAH-CHD duration (Table 5).

Additional significant correlations were observed between RV_SV_3D and RV_Ld_3D was negatively correlated with LV_AIS (partial r = −0.85, *p* = 0.0016, *p*-corrected = 0.0352) (Table 5).

### 3.4. Correlations Between 3D RV Indices and LA and RA Strain Indices

The negative linear correlation of RV_TAPSE_3D with RASct_ED and RASct_AC remained significant after controlling for PAH-CHD duration (Table 6).

## 4. Discussion

The present study provides an integrated three-dimensional and deformation-based characterization of cardiac remodeling in children with PAH-CHD. The main findings were that: (1) children with PAH-CHD exhibited marked RV remodeling, characterized by increased 3D RV volumes and transverse dimensions, reduced longitudinal dimension, and impaired systolic performance; (2) most 3DE-derived RV indices showed significant ability to discriminate PAH-CHD from healthy controls, with RV_FAC_3D and RV_EF_3D demonstrating good significant discriminative performance; (3) 3D measures of RV function and remodeling were significantly correlated with selected conventional RV functional and diastolic indices; and (4) strong relationships between RV geometry and LV volumes, mass, and selected regional deformation indices supported the presence of coordinated biventricular remodeling. In contrast, correlations with atrial deformation were limited. Taken together, these findings extend previous pediatric observations on 3DE-derived RV remodeling and dysfunction by integrating volumetric and geometric assessment with conventional functional indices and biventricular and atrial deformation in children with PAH-CHD [29,30,31]. The contribution of this study lies in this comprehensive characterization of cardiac remodeling and the exploratory relationships among these measures, rather than in demonstrating diagnostic superiority or incremental clinical value of 3DE.

The PAH-CHD cohort represented a clinically affected pediatric population, with all patients classified as WHO-FC II or III and a substantially reduced 6MWT distance compared with healthy controls. Most patients had Eisenmenger physiology, predominantly related to VSD, whereas the remaining patients had persistent or recurrent PAH following surgical repair. This distribution reflects the heterogeneous anatomical substrates through which CHD may lead to advanced pulmonary vascular disease [1,2,3]. These clinical subgroups differ in their underlying hemodynamic characteristics and patterns of RV adaptation. In Eisenmenger syndrome, the duration and magnitude of RV pressure overload depend on the underlying congenital anatomy and shunt physiology, whereas persistent or recurrent PAH following defect repair may impose different loading conditions in the absence of the original intracardiac communication. These differences may influence RV volumes, geometry, and systolic function. Given the small sample size, subgroup analyses were not feasible, and the pooled findings should therefore be interpreted as reflecting a heterogeneous pediatric PAH-CHD population. Trisomy 21 was also frequent in our cohort, consistent with its recognized association with early pulmonary vascular disease, particularly in children with AVD [39]. Importantly, the groups were comparable in age, sex, BSA, and BMI, reducing the potential influence of differences in somatic growth on the observed echocardiographic phenotype. This is particularly relevant in pediatric 3DE, because RV volumes and dimensions vary substantially with body size and maturation [24,27,28].

### 4.1. Three-Dimensional Right Ventricular Remodeling and Systolic Function

The most prominent finding was the distinct 3D RV phenotype observed in children with PAH-CHD. RV_EDV_3D and RV_ESV_3D were increased, whereas RV_EF_3D and RV_FAC_3D were markedly reduced compared with controls. These findings are consistent with progressive RV adaptation to chronic pressure overload, in which increasing afterload promotes chamber remodeling and, as compensatory mechanisms become insufficient, deterioration of global systolic performance [1,2,3,21]. Previous studies in children with pulmonary hypertension have similarly demonstrated that 3DE identifies abnormal RV volumes and impaired systolic function and provides clinically relevant information beyond conventional echocardiographic indices [30,31].

Interestingly, RV_SV_3D was higher in children with PAH-CHD despite their lower RV_EF_3D. In the context of substantially increased end-diastolic volume, this finding may reflect a dilation-associated compensatory response that maintains absolute SV despite reduced fractional emptying. This observation should nevertheless be interpreted cautiously, because SV in PAH-CHD, particularly in patients with Eisenmenger physiology or residual shunting, cannot be considered equivalent to effective systemic forward flow.

The geometric changes identified by 3DE provide additional insight into the pattern of RV remodeling. Both basal and mid-cavity diameters were increased, whereas RV longitudinal dimension was reduced. Thus, RV remodeling in our cohort was not characterized simply by uniform chamber enlargement, but by a change in chamber configuration toward greater transverse expansion and reduced longitudinal dimension. This observation is particularly relevant given the complex three-dimensional nature of RV contraction. 3DE studies have shown that global RV ejection results from the interaction of longitudinal, radial, and anteroposterior shortening, with the relative contribution of these components varying with age and loading conditions [25,26]. The present findings therefore support evaluation of RV geometry and global function rather than reliance on longitudinal shortening alone.

The exploratory ROC analysis provided further evidence of between-group discrimination. Except for RV_TAPSE_3D and RV_SV_3D, all evaluated 3D RV indices significantly discriminated children with PAH-CHD from healthy controls after correction for multiple comparisons. RV_FAC_3D and RV_EF_3D showed the highest estimated AUC values, at 0.88 and 0.85, respectively, while RV basal and mid-cavity dimensions also demonstrated good discriminative performance. In contrast, RV_TAPSE_3D, despite being significantly lower in PAH-CHD at the group level, did not achieve statistically significant discrimination in the ROC analysis. TAPSE primarily reflects longitudinal annular displacement and therefore interrogates only one component of the complex RV contraction pattern [6,13,25]. Our results are consistent with the concept that integrative measures of chamber function and geometry may better characterize the heterogeneous mechanical response of the pressure-loaded RV [29,30].

The ROC-derived thresholds should not, however, be interpreted as clinically validated diagnostic cutoffs. They were derived from a small cohort and were intended to characterize the discrimination achieved by individual 3D parameters within the present study population. Although several 3DE-derived parameters demonstrated discrimination between children with established PAH-CHD and healthy controls, these findings do not establish diagnostic accuracy in children with suspected pulmonary hypertension or incremental value beyond conventional echocardiographic assessment. External validation in larger, clinically representative pediatric cohorts would therefore be required before these thresholds could be considered for clinical decision-making. Similarly, although an initial difference was observed between the ROC curves for RV_EDV_3D and RV_ESV_3D, this difference did not remain significant after correction for multiple comparisons and should not be interpreted as evidence of superiority of one volumetric measure over the other.

### 4.2. Relationship Between 3D Right Ventricular Indices and Conventional Right Ventricular Assessment

The relationships between 3DE-derived indices and conventional echocardiographic parameters further illustrate the complementary nature of these approaches. RV_FAC_2D was strongly correlated with RV_EF_3D and RV_FAC_3D, supporting internal consistency between area-based and volumetric measures of global RV systolic performance. By contrast, TAPSE_2D was not significantly associated with the evaluated 3D indices. This finding again emphasizes that longitudinal annular excursion and global RV performance are not interchangeable, particularly when RV geometry is substantially altered [6,13].

Several correlations were also observed between 3D RV remodeling and tissue Doppler-derived diastolic indices. Lower Tr_E′ was associated with larger RV volumes and selected RV dimensions, whereas higher Tr_E/E′ was associated with greater RV volumes and transverse dimensions. These relationships suggest that structural remodeling and abnormalities in RV diastolic mechanics coexist in PAH-CHD. However, tissue Doppler indices are load-dependent, and the interpretation of E/E′ as a direct surrogate of filling pressure is less established for the RV than for the LV. These findings should therefore be regarded as evidence of an association between RV remodeling and altered diastolic function rather than as direct evidence of increased RV filling pressures [6,13]. Taken together, these findings highlight the complementary role of 3DE in characterizing RV volumes, global systolic function, and geometric remodeling alongside conventional echocardiographic indices. However, the present study does not establish the incremental diagnostic value of 3DE over conventional assessment.

Correlations between 3D RV measures and RV longitudinal strain were comparatively limited. Neither RV free-wall longitudinal strain nor four-chamber longitudinal strain showed significant associations with the principal 3D RV indices, while a significant relationship was observed only between mid-RV segmental strain and RV mid-cavity diameter. This should not be interpreted as evidence that deformation imaging and 3DE assess unrelated processes. Rather, the two techniques interrogate different aspects of RV performance: longitudinal strain primarily reflects myocardial deformation along the longitudinal axis, whereas 3DE captures global chamber geometry, volumetric remodeling, and the integrated result of multiple components of RV contraction [16,22,23,30,31]. In a small cohort, these complementary measures may therefore not demonstrate consistent linear relationships despite reflecting different manifestations of the same disease process.

### 4.3. Biventricular Remodeling and Ventricular Interdependence

One of the most notable secondary findings was the strong relationship between 3D RV remodeling and LV geometry. RV_EDV_3D and RV_ESV_3D correlated strongly with LV_ED_mass_3D, and RV_SV_3D was closely correlated with LV volumes and LV_ED_mass_3D; RV_EF_3D was similarly correlated with LV_EF_3D. These findings support the view that the cardiovascular consequences of PAH extend beyond the right ventricle and involve coordinated biventricular adaptation.

Ventricular interdependence is particularly relevant in pulmonary hypertension, since the two ventricles share the interventricular septum and are constrained within the pericardial space. Progressive RV pressure overload and remodeling can shift septal position, alter LV geometry, and impair LV filling, affecting LV mechanics even without primary left-sided disease [35]. Previous pediatric studies have linked pulmonary hemodynamic abnormalities and RV pressure overload to altered LV diastolic function, supporting routine LV assessment in children with pulmonary hypertension [35,36]. The linear correlations observed here between RV volumes and LV mass and volumes extend this concept to three-dimensional chamber remodeling.

Regional LV deformation offered a further, exploratory signal of ventricular interaction. Apical inferoseptal strain was associated with RV_SV_3D, RV_Ld_3D, and RV_TAPSE_3D, while global LV longitudinal strain showed no significant correlation with any 3D RV index. Given the direct mechanical involvement of the interventricular septum in RV-LV interaction, an association with regional inferoseptal deformation is physiologically plausible. The lack of association with global strain, together with the number of segmental comparisons performed, means this pattern cannot be taken as evidence that ventricular interdependence in PAH-CHD is predominantly regional; it is better regarded as hypothesis-generating and warrants confirmation in larger, purpose-designed cohorts.

Selected LV diastolic indices were also correlated with RV systolic and geometric measures: RV_FAC_3D correlated with Mi_E′ and Mi_E/E′, and RV_EF_3D and RV_Dd_mid_3D with Mi_IVRT, although not all correlations remained significant after correction for multiple comparison. Together with the volumetric findings, these observations support the view that RV remodeling in PAH-CHD occurs within an integrated biventricular system rather than as an isolated right-sided process. Because all measurements were obtained at a single time point, however, the present data do not allow the direction or causal sequence of these relationships to be established.

### 4.4. Atrial Mechanics

In contrast to the broader pattern of ventricular associations, relationships between 3D RV indices and atrial deformation were limited. No LA strain component was significantly correlated with 3D RV measures, whereas RA contractile strain was negatively correlated with RV_TAPSE_3D using both end-diastolic and atrial-contraction reference points. Previous studies have demonstrated abnormalities in RA deformation in pediatric pulmonary hypertension and have suggested that atrial mechanics may provide information regarding disease severity and clinical outcome [33,34].

The selective negative associations between RA contractile strain and RV_TAPSE_3D may suggest an interaction between atrial deformation and longitudinal RV mechanics. However, the absence of corresponding associations with RV volumes, RV_EF_3D, or RV_FAC_3D limits their physiological interpretation. Given the small sample size and the number of correlations examined, these findings should be considered exploratory and do not establish a compensatory atrial mechanism. Further studies integrating RA deformation, RV mechanics, and invasive hemodynamics are needed to clarify their potential relationships in pediatric PAH-CHD.

Clinical Implications

Our findings support the integration of 3DE into a multiparametric assessment of RV function in children with PAH-CHD. While conventional longitudinal indices remain clinically useful, 3DE provides complementary information on RV volumes, global systolic function, and chamber geometry. In particular, RV_EF_3D and RV_FAC_3D showed acceptable-to-good discriminative performance, supporting the added value of global 3D assessment in characterizing RV remodeling [24,27,28,29,30].

Because all participants were assessed at a single time point, our study cannot characterize the longitudinal evolution of RV remodeling. In adults with end-stage renal disease undergoing dialysis, Arcari et al. reported progressive RV dilation and deterioration of RV systolic function on serial echocardiography [40]. Although the underlying disease and RV loading conditions differ substantially from those in pediatric PAH-CHD, these findings illustrate the value of longitudinal RV assessment. Future prospective studies should investigate whether serial 3DE-derived measurements, alongside conventional echocardiography and myocardial deformation imaging, can provide clinically relevant information on disease progression and treatment response in children with PAH-CHD.

Study Limitations

This study has several limitations. The small, single-center PAH-CHD cohort limited statistical precision and precluded meaningful subgroup analyses. Clinical and anatomical heterogeneity, including both Eisenmenger syndrome and persistent or recurrent PAH following CHD repair, may have introduced differences in RV loading conditions and remodeling patterns, further limiting the generalizability of our findings. Given the small sample size and the number of correlations examined, particularly for regional deformation parameters, these associations should be considered exploratory and hypothesis-generating. The single time-point assessment precludes evaluation of temporal changes and prognostic implications, while the ROC-derived thresholds require external validation before clinical application. Given the small PAH-CHD cohort, the resulting uncertainty in cutoff estimation, and the absence of internal or external validation, the ROC-derived thresholds should be considered exploratory rather than clinically validated diagnostic cutoffs. In addition, the thresholds were derived from a small sample with a wide age range and require validation in age-specific population.

In addition, all echocardiographic acquisitions and analyses were performed by a single experienced investigator using a standardized protocol; however, intra- and inter-observer reproducibility was not assessed. This limitation is particularly relevant for 3DE-derived RV measurements and speckle-tracking strain, which are influenced by endocardial delineation, tracking quality, and manual contour adjustments. The frequency of manual contour corrections was not systematically recorded, further limiting the assessment of measurement reproducibility. Future studies should incorporate formal reproducibility assessments to establish measurement reliability. Furthermore, RA deformation was assessed using an LA-specific software (QLAB 15) template, which may introduce measurement uncertainty, particularly in children with altered RA geometry. The resulting RA strain associations should therefore be interpreted with caution. Finally, 3DE remains dependent on image quality and complete RV visualization, and multi-beat acquisitions are susceptible to respiratory and stitching artifacts [24,27,28]. The lack of pediatric reference z-scores for the 3D RV volumetric parameters limited body-size normalization, which relied on BSA indexing and statistical adjustment. The small sample size limited the number of covariates that could be included in the partial correlation analyses and residual confounding by factors such as age, sex, BSA cannot be excluded. Larger prospective, multicenter studies are warranted to validate these findings and determine the longitudinal and prognostic relevance of 3DE-derived RV measures in pediatric PAH-CHD.

## 5. Conclusions

In children with PAH-CHD, 3DE revealed a coherent pattern of RV remodeling: chamber dilation, reduced ejection performance, and a shift toward a more globular geometry. Most evaluated 3D RV indices demonstrated between-group discrimination, except for RV_TAPSE_3D. RV volumetric and functional indices correlated with conventional 2D and diastolic Doppler parameters, LV geometry and segmental mechanics, and selected RA strain measures, suggesting potential biventricular interactions in this population. These exploratory findings suggest that 3DE may complement conventional echocardiography in the multiparametric assessment of pediatric PAH-CHD. Given the small, heterogeneous PAH-CHD cohort and single time-point assessment, the findings should be interpreted cautiously. The ROC-derived thresholds require independent validation in larger, clinically representative pediatric cohorts, and the incremental diagnostic and prognostic value of 3DE remains to be established.

## Figures and Tables

**Table 1:** Demographic, anthropometric and clinical characteristics of the studied groups.

	Control Group (*n*_1_ = 50)	PAH-CHD Group (*n*_2_ = 11)	*p*-Value
Age at study included (years), mean (SD)	10.70 (3.59)	11.73 (2.15)	0.3683
Sex (male), *n* (%)	33 (66)	5 (45.5)	0.3034
BSA (m^2^), mean (SD)	1.27 (0.33)	1.26 (0.25)	0.8985
BMI (kg/m^2^), median [IQR]	17.62 [16.09, 19.56]	17.28 [15.02, 18.93]	0.6059
HR (beats/min), mean (SD)	78.18 (7.29)	93 (12.84)	0.0033 *
sBP (mmHg), mean (SD)	109.72 (9.06)	111.73 (9.83)	0.5148
dBP (mmHg), mean (SD)	62.22 (6.15)	70.64 (5.55)	0.0001 *
Baseline WHO-FC, *n* (%)			
I		0 (0)	
II		5 (45.45)	
III		6 (54.55)	
IV		0 (0)	
Eisenmenger syndrome, *n* (%)			
VSD		5 (45.45)	
AVD		1 (9.09)	
VSD + ASD + PDA		1 (9.09)	
Postoperative PAH, *n* (%)			
AVD		1 (9.09)	
TGA		1 (9.09)	
PDA		1 (9.09)	
TGA + VSD		1 (9.09)	
Time since diagnosis (years), mean (SD)		3.82 (3.40)	
*Comorbidities, n (%)*			
Down syndrome		5 (45.45)	
hyperuricemia		2 (18.18)	
polyglobulia		7 (63.64)	
iron deficiency		5 (45.45)	
hepatopathy		5 (45.45)	
*Symptomatology, n (%)*			
fatigue		11 (100)	
dyspnea at rest		6 (54.55)	
exertional dyspnea		9 (81.82)	
orthopnea		1 (9.09)	
syncope		2 (18.18)	
cyanosis		8 (72.73)	
cough		2 (18.18)	
chest pain		2 (18.18)	
palpitations		1 (9.09)	
clinical signs of hyperviscosity syndrome		3 (27.27)	
failure to thrive		3 (27.27)	
*Medications, n (%)*			
Bosentan		3 (27.27)	
Sildenafil + Bosentan		8 (72.73)	
Initial monotherapy		7 (63.64)	
Initial combination therapy		4 (36.36)	
Changes in treatment		4 (36.36)	
*Baseline functional parameters*			
6MWT_distance (m)	631 (62.54)	406.55 (91.90)	0.000005 *
SaO2_6MWT (%)	100 [100, 100]	90 [89, 94]	<0.0001 *
HR_6MWT (beats/min)	100 [100, 100]	90 [81, 98]	<0.0001 *

PAH-CHD: pulmonary arterial hypertension associated with congenital heart disease; SD: standard deviation; BSA (m^2^): body surface area; BMI (kg/m^2^): body mass index; IQR: interquartile range; sBP (mmHg): systolic blood pressure; dBP (mmHg): diastolic blood pressure; HR: heart rate; WHO-FC: World Health Organization functional class; VSD: ventricular septal defect; AVD: atrioventricular septal defect; ASD: atrial septal defect; PDA: patent ductus arteriosus; TGA: transposition of the great arteries; 6MWT: six-minute walk test; SaO_2_: peripheral oxygen saturation; *n*: number; * Statistically significant, *p* < 0.05.

**Table 2:** Distributions of right ventricular functional indices between control and PAH-CHD groups.

	Control Group (*n*_1_ = 50)	PAH-CHD Group (*n*_2_ = 11)	Adjusted *p*-Value
RV_EDV_3D (mL)	84.64 (32.52)	119.79 (33.88)	0.0001 *
RV_ESV_3D (mL)	38.02 (16.82)	63.49 (20.10)	<0.0001 *
RV_EF_3D (%)	56.45 (4.82)	47.57 (6.48)	<0.0001 *
RV_SV_3D (mL)	46.62 (17.51)	56.30 (16.47)	0.0149 *
RV_TAPSE_3D (mm)	20.58 (3.11)	16.75 (5.40)	0.0279 *
RV_FAC_3D (%)	50.37 (5.18)	39.82 (6.97)	<0.0001 *
RV_Dd_base_3D (mm)	37.18 (7.13)	47.47 (6.93)	<0.0001 *
RV_Dd_mid_3D (mm)	34.25 (7.77)	46.05 (7.24)	<0.0001 *
RV_Ld_3D (mm)	69.72 (11.30)	80.06 (11.05)	0.0005 *

Data were presented as mean (SD); SD: standard deviation; PAH-CHD: pulmonary arterial hypertension associated with congenital heart disease; RV: right ventricle; RV_EDV_3D (mL): right ventricle end-diastolic volume measured by 3D echocardiography; RV_ESV_3D (mL): right ventricle end-systolic volume measured by 3D echocardiography; RV_EF_3D (%): right ventricle ejection fraction measured by 3D echocardiography; RV_SV_3D (mL): right ventricle stroke volume measured by 3D echocardiography; RV_TAPSE_3D (mm): tricuspid annular plane systolic excursion obtained by 3D echocardiography; RV_FAC_3D (%): right ventricle fractional area change obtained by 3D echocardiography; RV_Dd_base_3D (mm): right ventricle basal diameter measured at the basal third of the right ventricle in end-diastole by 3D echocardiography; RV_Dd_mid_3D (mm): right ventricle mid-cavity diameter measured at the mid-ventricular level of the right ventricle in end-diastole by 3D echocardiography; RV_Ld_3D (mm): right ventricle longitudinal distance from the tricuspid annulus to the right ventricle apex in end-diastole measured by 3D echocardiography; *p*-values were derived from Type-III ANCOVA models, adjusted for BSA z-score; * Significant result, *p* < 0.05.

**Table 3:** Area under curve (AUC) estimates of right ventricular functional indices obtained by 3D echocardiography.

	AUC	95% CI	*p*	*p*- Corrected	Cutoff	Direction	Se (%) [95% CI]	Sp (%) [95% CI]
RV_EDV_3D (mL)	0.78	[0.65, 0.91]	0.00004 *	0.00006 *	82.35	↑ cutoff: PAH-CHD	100 [72.73, 100]	54 [42, 90]
RV_ESV_3D (mL)	0.84	[0.73, 0.95]	<0.0001 *	<0.0001 *	40.45	↑ cutoff: PAH-CHD	100 [72.73, 100]	60 [50, 96]
RV_EF_3D (%)	0.85	[0.70, 1]	<0.0001 *	0.00001 *	49.9	↓ cutoff: PAH-CHD	72.73 [39.03, 93.98]	100 [92.89, 100]
RV_SV_3D (mL)	0.68	[0.51, 0.85]	0.0447 *	0.0503	49.6	↑ cutoff: PAH-CHD	72.73 [36.36, 100]	62 [20, 94]
RV_TAPSE_3D (mm)	0.71	[0.49, 0.93]	0.0596	0.0596	15.65	↓ cutoff: PAH-CHD	54.55 [27.27, 90.91]	96 [67.9, 100]
RV_FAC_3D (%)	0.88	[0.73, 1]	<0.0001 *	<0.0001 *	40.2	↓ cutoff: PAH-CHD	72.73 [54.55, 100]	100 [82, 100]
RV_Dd_base_3D (mm)	0.86	[0.76, 0.97]	<0.0001 *	<0.0001 *	40.3	↑ cutoff: PAH-CHD	90.9 [63.6, 100]	72 [52, 96]
RV_Dd_mid_3D (mm)	0.86	[0.80, 0.97]	<0.0001 *	<0.0001 *	37.55	↑ cutoff: PAH-CHD	100 [81.82, 100]	74 [62, 94]
RV_Ld_3D (mm)	0.75	[0.60, 0.91]	0.0014 *	0.0018 *	80.25	↑ cutoff: PAH-CHD	54.55 [45.45, 100]	86 [34, 98]

AUC: Area under curve; 95% CI: 95% confidence interval; Se: sensibility; Sp: specificity; * significant result: *p* < 0.05; *p*-values were calculated using a Wald test based on the DeLong variance estimate of the AUC; *p*-corrected for multiple comparisons using Benjamini–Hochberg method; cutoff values were determined using the Youden index and have not undergone internal or external validation; they should therefore be considered exploratory. PAH-CHD: pulmonary arterial hypertension associated with congenital heart disease; RV: right ventricle; RV_EDV_3D (mL): right ventricle end-diastolic volume measured by 3D echocardiography; RV_ESV_3D (mL): right ventricle end-systolic volume measured by 3D echocardiography; RV_EF_3D (%): right ventricle ejection fraction measured by 3D echocardiography; RV_SV_3D (mL): right ventricle stroke volume measured by 3D echocardiography; RV_TAPSE_3D (mm): tricuspid annular plane systolic excursion obtained by 3D echocardiography; RV_FAC_3D (%): right ventricle fractional area change obtained by 3D echocardiography; RV_Dd_base_3D (mm): right ventricle basal diameter measured at the basal third of the right ventricle in end-diastole by 3D echocardiography; RV_Dd_mid_3D (mm): right ventricle mid-cavity diameter measured at the mid-ventricular level of the right ventricle in end-diastole by 3D echocardiography; RV_Ld_3D (mm): right ventricle longitudinal distance from the tricuspid annulus to the right ventricle apex in end-diastole measured by 3D echocardiography.

**Table 4:** Partial Pearson correlation coefficients between right ventricular parameters in the PAH-CHD group.

	RV_EDV_3D (mL)	RV_ESV_3D (mL)	RV_EF_3D (%)	RV_SV_3D (mL)	RV_TAPSE_3D (mm)	RV_FAC_3D (%)	RV_Dd_Base_3D (mm)	RV_Dd_Mid_3D (mm)	RV_Ld_3D (mm)
Conventional echocardiographic metrics									
TAPSE_2D (mm)	−0.48 (0.1562)	−0.34 (0.3426)	−0.02 (0.9526)	−0.53 (0.1165)	0.37 (0.2885)	−0.17 (0.6388)	0.15 (0.6722)	−0.27 (0.4566)	−0.06 (0.8787)
RV_FAC_2D (%)	−0.01 (0.9984)	−0.36 (0.3060)	0.88 (0.0007 *^#^)	0.48 (0.1577)	0.45 (0.1462)	0.89 (0.0005 *^#^)	−0.05 (0.8859)	−0.62 (0.0554)	0.01 (0.9825)
RV_Tei_2D	0.26 (0.4691)	0.09 (0.7924)	0.34 (0.3299)	0.40 (0.2583)	0.60 (0.0679)	0.10 (0.7867)	0.01 (0.9693)	−0.11 (0.7867)	0.35 (0.3212)
Tr_E/A	0.26 (0.4626)	0.28 (0.4285)	−0.38 (0.2779)	0.15 (0.6782)	−0.56 (0.0892)	0.17 (0.6349)	−0.15 (0.6591)	0.23 (0.5157)	−0.16 (0.6591)
Tr_E′ (cm/s)	−0.81 (0.0047 *^#^)	−0.76 (0.0099 *^#^)	0.09 (0.8114)	−0.66 (0.0384 *)	−0.25 (0.4787)	0.10 (0.9798)	−0.68 (0.0312 *)	−0.57 (0.0827)	−0.81 (0.0042 *^#^)
Tr_A′ (cm/s)	−0.29 (0.4173)	−0.06 (0.8597)	−0.22 (0.5355)	−0.52 (0.1253)	−0.03 (0.9428)	−0.44 (0.2028)	−0.16 (0.6669)	0.10 (0.7799)	0.14 (0.6991)
Tr_S (cm/s)	−0.33 (0.3481)	−0.24 (0.5049)	0.21 (0.5703)	−0.36 (0.3050)	0.33 (0.3440)	−0.31 (0.3828)	−0.12 (0.7514)	−0.17 (0.6337)	0.13 (0.7211)
Tr_E/E′	0.82 (0.0037 *^#^)	0.82 (0.0033 *^#^)	−0.23 (0.5297)	0.67 (0.0346 *)	−0.12 (0.7354)	−0.04 (0.9134)	0.81 (0.0049 *)	0.74 (0.0141 *)	0.49 (0.1544)
Tr_IVCT (ms)	0.37 (0.2870)	0.47 (0.1703)	−0.47 (0.1765)	0.20 (0.5734)	−0.15 (0.6882)	−0.35 (0.3228)	0.71 (0.0218 *^#^)	0.54 (0.1088)	0.19 (0.6051)
Tr_IVRT (ms)	0.36 (0.3136)	0.34 (0.3312)	−0.24 (0.4992)	0.28 (0.4243)	−0.13 (0.7273)	−0.10 (0.8051)	0.34 (0.3413)	0.40 (0.2568)	0.31 (0.3847)
Strain parameters									
BRV (%)	0.16 (0.6648)	0.15 (0.6731)	0.01 (0.9678)	0.38 (0.2827)	0.27 (0.4432)	−0.14 (0.7004)	−0.001 (0.9804)	0.18 (0.6137)	0.32 (0.3660)
MRV (%)	0.38 (0.2811)	0.58 (0.0807)	−0.61 (0.0628)	0.22 (0.5491)	−0.14 (0.7006)	−0.38 (0.2804)	0.47 (0.1661)	0.67 (0.0333 *)	0.51 (0.1363)
ARV (%)	0.27 (0.4551)	0.44 (0.1981)	−0.45 (0.1893)	0.31 (0.3784)	0.12 (0.9744)	−0.36 (0.3138)	0.47 (0.1731)	0.55 (0.0992)	0.54 (0.1048)
RVFWSL (%)	0.29 (0.4192)	0.43 (0.2163)	−0.39 (0.2696)	0.33 (0.3457)	0.06 (0.8705)	−0.33 (0.3544)	0.34 (0.3367)	0.52 (0.1271)	0.51 (0.1327)
RV4CSL (%)	0.31 (0.3878)	0.45 (0.1952)	−0.44 (0.2031)	0.27 (0.4580)	−0.01 (0.9706)	−0.27 (0.4481)	0.41 (0.2420)	0.53 (0.1168)	0.48 (0.1618)

Values represented partial Pearson correlation coefficient and corresponding *p*-values, adjusted for PAH-CHD duration; * significant result, *p* < 0.05. ^#^ adjusted *p*-values that remained significant (<0.05) after the Benjamini–Hochberg procedure. RV: right ventricle; RV_EDV_3D (mL): right ventricle end-diastolic volume measured by 3D echocardiography; RV_ESV_3D (mL): right ventricle end-systolic volume measured by 3D echocardiography; RV_EF_3D (%): right ventricle ejection fraction measured by 3D echocardiography; RV_SV_3D (mL): right ventricle stroke volume measured by 3D echocardiography; RV_TAPSE_3D (mm): tricuspid annular plane systolic excursion obtained by 3D echocardiography; RV_FAC_3D (%): right ventricle fractional area change obtained by 3D echocardiography; RV_Dd_base_3D (mm): right ventricle basal diameter measured at the basal third of the right ventricle in end-diastole by 3D echocardiography; RV_Dd_mid_3D (mm): right ventricle mid-cavity diameter measured at the mid-ventricular level of the right ventricle in end-diastole by 3D echocardiography; RV_Ld_3D (mm): right ventricle longitudinal distance from the tricuspid annulus to the right ventricle apex in end-diastole measured by 3D echocardiography; TAPSE_2D (mm): tricuspid annular plane systolic excursion obtained by 2D echocardiography; RV_FAC_2D (%): right ventricle fractional area change obtained by 2D echocardiography; RV_Tei_2D: right ventricular myocardial performance index; Tr_E/A: ratio between early tricuspid inflow velocity and late tricuspid inflow velocity; Tr_E′ (cm/s): tricuspid annular early diastolic velocity (E′ wave) obtained by tissue Doppler echocardiography; Tr_A′ (cm/s): tricuspid annular late diastolic velocity (A′ wave) obtained by tissue Doppler echocardiography; Tr_S (cm/s): tricuspid annular systolic velocity (S wave) obtained by tissue Doppler echocardiography; Tr_E/E′: ratio between early tricuspid inflow velocity and tricuspid annular early diastolic velocity (E/E′); Tr_IVCT (ms): right ventricle isovolumic contraction time; Tr_IVRT (ms): right ventricle isovolumic relaxation time; BRV (%): basal right ventricle segment longitudinal strain; MRV (%): mid right ventricle segment longitudinal strain; ARV (%): apical right ventricle segment longitudinal strain; RVFWSL (%): right ventricle free wall longitudinal strain (autostrain); RV4CSL (%): right ventricle apical four-chamber view total strain (autostrain).

**Table 5:** Partial Pearson correlation coefficients between 3D RV indices and left ventricular parameters in the PAH-CHD group.

	RV_EDV_3D (mL)	RV_ESV_3D (mL)	RV_EF_3D (%)	RV_SV_3D (mL)	RV_TAPSE_3D (mm)	RV_FAC_3D (%)	RV_Dd_Base_3D (mm)	RV_Dd_Mid_3D (mm)	RV_Ld_3D (mm)
Conventional 2D echocardiographic parameters									
MAPSE (mm)	0.66 (0.0384 *)	0.53 (0.1116)	0.02 (0.9652)	0.67 (0.0330 *)	−0.01 (0.9819)	0.03 (0.9264)	0.32 (0.3670)	0.46 (0.1794)	0.22 (0.5334)
Mi_E/A	0.49 (0.1499)	0.21 (0.5581)	0.44 (0.2066)	0.758 (0.0118 * 0.0)	0.33 (0.3553)	0.63 (0.0498 *)	0.49 (0.1505)	−0.005 (0.5893)	0.44 (0.1960)
Mi_E′ (cm/s)	−0.07 (0.8430)	−0.30 (0.3934)	0.39 (0.2656)	0.24 (0.513)	0.03 (0.9426)	0.72 (0.0198 *)	−0.27 (0.4477)	−0.41 (0.2388)	−0.16 (0.6616)
Mi_A′ (cm/s)	−0.26 (0.4768)	−0.10 (0.7798)	−0.11 (0.7645)	−0.49 (0.1506)	−0.02 (0.9473)	−0.26 (0.4627)	0.15 (0.6802)	0.05 (0.8812)	−0.33 (0.3479)
Mi_S (cm/s)	0.22 (0.5482)	0.15 (0.6889)	0.06 (0.8635)	0.25 (0.4773)	−0.13 (0.7241)	0.35 (0.3224)	−0.21 (0.5533)	0.10 (0.7897)	−0.06 (0.8706)
Mi_E/E′	0.22 (0.5387)	0.47 (0.1701)	−0.49 (0.1468)	−0.14 (0.6975)	−0.11 (0.7651)	−0.65 (0.0435 *)	0.52 (0.1257)	0.60 (0.0673)	0.37 (0.2919)
Mi_IVCT (ms)	−0.05 (0.8926)	0.11 (0.7614)	−0.52 (0.1204)	−0.25 (0.4864)	−0.23 (0.5155)	−0.55 (0.0977)	0.19 (0.6049)	0.21 (0.5525)	−0.12 (0.7329)
Mi_IVRT (ms)	0.49 (0.1481)	0.60 (0.0663)	−0.67 (0.0335 *)	0.22 (0.5450)	−0.45 (0.1871)	−0.32 (0.3596)	0.40 (0.2523)	0.66 (0.0375 *)	0.21 (0.5559)
3D LV echocardiographic parameters									
LV_EDV_3D (mL)	0.63 (0.0511)	0.42 (0.2325)	0.48 (0.1640)	0.73 (0.0165)	0.62 (0.0574)	0.26 (0.4647)	0.36 (0.3064)	0.15 (0.6796)	0.85 (0.0018 *^#^)
LV_ESV_3D (mL)	0.35 (0.3190)	0.28 (0.4369)	0.31 (0.3861)	0.38 (0.2837)	0.58 (0.07921)	0.22 (0.5316)	0.45 (0.1865)	0.15 (0.6882)	0.79 (0.0067 *^#^)
LV_EF_3D (%)	−0.11 (0.7542)	−0.36 (0.3056)	0.64 (0.0457 *)	0.20 (0.5718)	0.25 (0.4862)	0.32 (0.3631)	−0.38 (0.2841)	−0.55 (0.1018)	−0.21 (0.5655)
LV_EDL_3D (mm)	0.42 (0.2292)	0.50 (0.1380)	−0.24 (0.4962)	0.22 (0.5438)	0.19 (0.5916)	0.02 (0.9659)	0.25 (0.4876)	0.45 (0.1948)	0.54 (0.1057)
LV_ESL_3D (mm)	0.42 (0.2255)	0.43 (0.2099)	−0.05 (0.9010)	0.35 (0.3154)	0.35 (0.3249)	0.26 (0.4655)	0.53 (0.1162)	0.35 (0.3173)	0.59 (0.0720)
LV_SV_3D (mL)	0.43 (0.2093)	0.44 (0.2060)	−0.25 (0.4784)	0.26 (0.4637)	−0.05 (0.8854)	−0.04 (0.9058)	−0.23 (0.5241)	0.33 (0.3592)	0.26 (0.4766)
LV_ED_mass_3D (g)	0.89 (0.0006 *)	0.85 (0.0017 *)	−0.13 (0.7178)	0.71 (0.0206 *)	−0.06 (0.8689)	0.15 (0.6841)	0.51 (0.1328)	0.72 (0.0194 *)	0.76 (0.0101 *^#^)
Strain metrics									
LV_BIS (%)	−0.35 (0.3236)	−0.22 (0.5495)	−0.32 (0.3676)	−0.41 (0.2350)	−0.39 (0.2648)	0.02 (0.9511)	−0.05 (0.9017)	−0.01 (0.9846)	−0.32 (0.3685)
LV_MIS (%)	−0.01 (0.9678)	0.22 (0.5435)	−0.62 (0.0543)	−0.26 (0.4645)	−0.40 (0.2562)	0.002 (0.9946)	0.24 (0.5035)	0.31 (0.3884)	−0.11 (0.7555)
LV_AIS (%)	−0.55 (0.0981)	−0.38 (0.2819)	−0.43 (0.2173)	−0.59 (0.0696)	−0.71 (0.0227 *)	−0.13 (0.7232)	−0.24 (0.5021)	−0.12 (0.7317)	−0.85 (0.0016 *^#^)
LV_BAL (%)	−0.31 (0.3837)	−0.51 (0.1295)	0.49 (0.1544)	−0.07 (0.8581)	0.23 (0.5173)	0.27 (0.4534)	−0.73 (0.0182 *)	−0.66 (0.0375 *)	−0.13 (0.7123)
LV_MAL (%)	−0.41 (0.2416)	−0.20 (0.5707)	−0.37 (0.2886)	−0.57 (0.0828)	0.01 (0.9886)	−0.31 (0.3794)	0.13 (0.7278)	−0.06 (0.8589)	−0.01 (0.9816)
LV_AAL (%)	−0.22 (0.5329)	−0.15 (0.4726)	−0.23 (0.5300)	−0.19 (0.6080)	−0.64 (0.0457 *)	−0.09 (0.8149)	0.02 (0.9518)	0.06 (0.8693)	−0.63 (0.0511)
LV_BI (%)	−0.02 (0.9546)	−0.20 (0.5858)	0.35 (0.3256)	0.16 (0.6503)	−0.18 (0.6198)	0.36 (0.3137)	−0.53 (0.1111)	−0.26 (0.4632)	−0.03 (0.9281)
LV_MI (%)	−0.24 (0.5026)	−0.27 (0.4592)	0.30 (0.3957)	−0.15 (0.6763)	0.17 (0.6342)	0.22 (0.5383)	−0.34 (0.3379)	−0.24 (0.4970)	0.06 (0.8633)
LV_AI (%)	−0.23 (0.5261)	−0.07 (0.8456)	0.15 (0.6899)	−0.34 (0.3408)	0.18 (0.6225)	0.11 (0.7538)	0.16 (0.6560)	−0.05 (0.8910)	0.17 (0.6452)
LV_BA (%)	−0.48 (0.1635)	−0.44 (0.2075)	0.21 (0.5673)	−0.44 (0.2058)	0.39 (0.2668)	0.01 (0.9693)	−0.21 (0.5596)	−0.40 (0.2585)	0.17 (0.6295)
LV_MA (%)	0.32 (0.3712)	0.44 (0.2061)	−0.18 (0.6158)	0.10 (0.7710)	−0.20 (0.5738)	−0.21 (0.5563)	0.15 (0.6872)	0.51 (0.1351)	0.30 (0.3970)
LV_AA (%)	−0.16 (0.6548)	−0.12 (0.7366)	0.03 (0.9348)	−0.14 (0.6955)	−0.15 (0.6827)	0.22 (0.5444)	−0.05 (0.8821)	−0.02 (0.9464)	−0.01 (0.9794)
LV_BIL (%)	0.07 (0.8439)	0.07 (0.8372)	−0.14 (0.7046)	−0.05 (0.8870)	0.08 (0.8368)	−0.43 (0.2124)	−0.36 (0.3111)	0.03 (0.9316)	0.21 (0.5533)
LV_MIl (%)	−0.57 (0.0853)	−0.37 (0.2995)	−0.27 (0.4441)	−0.70 (0.0251 *)	−0.29 (0.4150)	−0.27 (0.4519)	−0.34 (0.3438)	−0.11 (0.7537)	−0.31 (0.3897)
LV_AL (%)	−0.36 (0.3099)	−0.22 (0.5458)	−0.21 (0.5682)	−0.38 (0.2781)	−0.07 (0.8495)	0.11 (0.7664)	0.19 (0.6079)	−0.04 (0.9042)	−0.21 (0.5528)
LV_BAS (%)	−0.19 (0.6010)	−0.07 (0.8556)	−0.32 (0.3646)	−0.33 (0.3535)	−0.09 (0.7951)	−0.04 (0.9063)	−0.01 (0.9698)	−0.10 (0.7889)	−0.08 (0.8164)
LV_MAS (%)	0.08 (0.8319)	0.17 (0.6354)	−0.28 (0.4280)	−0.09 (0.7977)	−0.04 (0.9215)	−0.02 (0.9513)	−0.19 (0.5972)	0.20 (0.5749)	0.23 (0.5300)
LV_AA* (%)	−0.33 (0.3549)	−0.32 (0.3596)	0.08 (0.8187)	−0.26 (0.4678)	0.24 (0.5089)	0.18 (0.6155)	−0.32 (0.3652)	−0.28 (0.4333)	−0.04 (0.9124)
LV_GLS_A4C (%)	−0.53 (0.1135)	−0.47 (0.1733)	−0.17 (0.6427)	−0.46 (0.1802)	−0.40 (0.2518)	0.10 (0.7773)	−0.19 (0.6073)	−0.30 (0.3990)	−0.53 (0.1181)
LV_GLS_A2C (%)	0.02 (0.9623)	0.16 (0.6569)	−0.20 (0.5747)	−0.20 (0.5917)	−0.12 (0.7401)	−0.27 (0.4488)	−0.13 (0.7285)	0.27 (0.4445)	0.30 (0.3949)
LV_GLS_A3C (%)	−0.23 (0.5239)	−0.05 (0.8879)	−0.42 (0.2220)	−0.46 (0.1849)	−0.06 (0.8680)	−0.39 (0.2654)	−0.32 (0.3681)	0.07 (0.8388)	0.06 (0.8611)
LV_GLS (%)	−0.24 (0.5078)	−0.07 (0.8582)	−0.37 (0.2927)	−0.43 (0.2186)	−0.19 (0.6087)	−0.26 (0.4740)	−0.20 (0.5703)	0.10 (0.7920)	0.04 (0.9046)

RV: right ventricle; RV_EDV_3D (mL): right ventricle end-diastolic volume measured by 3D echocardiography; RV_ESV_3D (mL): right ventricle end-systolic volume measured by 3D echocardiography; RV_EF_3D (%): right ventricle ejection fraction measured by 3D echocardiography; RV_SV_3D (mL): right ventricle stroke volume measured by 3D echocardiography; RV_TAPSE_3D (mm): tricuspid annular plane systolic excursion obtained by 3D echocardiography; RV_FAC_3D (%): right ventricle fractional area change obtained by 3D echocardiography; RV_Dd_base_3D (mm): right ventricle basal diameter measured at the basal third of the right ventricle in end-diastole by 3D echocardiography; RV_Dd_mid_3D (mm): right ventricle mid-cavity diameter measured at the mid-ventricular level of the right ventricle in end-diastole by 3D echocardiography; RV_Ld_3D (mm): right ventricle longitudinal distance from the tricuspid annulus to the right ventricle apex in end-diastole measured by 3D echocardiography; MAPSE (mm): mitral annular plane systolic excursion; Mi_E/A: ratio between early and late mitral inflow velocity; Mi_E′ (cm/s): mitral annular early diastolic velocity (E′ wave); Mi_A′ (cm/s): mitral annular late diastolic velocity (A′ wave); Mi_S (cm/s): mitral annular systolic velocity (S wave); Mi_E/E′: ratio between early mitral inflow velocity and mitral annular early diastolic velocity; Mi_IVCT (ms): left ventricle isovolumic contraction time; Mi_IVRT (ms): left ventricle isovolumic relaxation time; LV: left ventricle; LV_EDV_3D (mL): left ventricle end-diastolic volume measured by 3D echocardiography; LV_ESV_3D (mL): left ventricle end-systolic volume measured by 3D echocardiography; LV_EF_3D (%): left ventricle ejection fraction measured by 3D echocardiography; LV_EDL_3D (mm): three-dimensional left ventricular end-diastolic length; LV_ESL_3D (mm): three-dimensional left ventricular end-systolic length; LV_SV_3D (mL): three-dimensional left ventricular stroke volume; LV_ED_mass_3D (g): three-dimensional left ventricular end-diastolic mass; LV_BIS (%): left ventricle basal inferoseptal strain; LV_MIS (%): left ventricle mid inferoseptal strain; LV_AIS (%): left ventricle apical inferoseptal strain; LV_BAL (%): left ventricle basal anterolateral strain; LV_MAL (%): left ventricle mid anterolateral strain; LV_AAL (%): left ventricle apical anterolateral strain; LV_BI (%): left ventricle basal inferior strain; LV_MI (%): left ventricle mid inferior strain; LV_AI (%): left ventricle apical inferior strain; LV_BA (%): left ventricle basal anterior strain; LV_MA (%): left ventricle mid anterior strain; LV_AA (%): left ventricle apical anterior strain (assessed from apical two chamber view); LV_BIL (%): left ventricle basal inferolateral strain; LV_MIL (%): left ventricle mid inferolateral strain; LV_AL (%): left ventricle apical lateral strain; LV_BAS (%): left ventricle basal anteroseptal strain; LV_MAS (%): left ventricle mid anteroseptal strain; LV_AA* (%): left ventricle apical anterior strain (assessed from apical three chamber view); LV_GLS_A4C (%): left ventricular apical four chamber longitudinal strain; LV_GLS_A2C (%): left ventricular apical two chamber longitudinal strain; LV_GLS_A3C (%): left ventricular apical three chamber longitudinal strain; LV_GLS (%): left ventricular global longitudinal strain (autostrain); * significant result, *p* < 0.05. ^#^ adjusted *p*-values that remained significant (<0.05) after the Benjamini–Hochberg procedure.

**Table 6:** Correlations between 3D RV indices and left atrium and right atrium strain parameters in the PAH-CHD group adjusted for disease duration.

	RV_EDV_3D (mL)	RV_ESV_3D (mL)	RV_EF_3D (%)	RV_SV_3D (mL)	RV_TAPSE_3D (mm)	RV_FAC_3D (%)	RV_Dd_Base_3D (mm)	RV_Dd_Mid_3D (mm)	RV_Ld_3D (mm)
LA (left atrium) strain metrics									
LASr_ED (%)	−0.52 (0.1190)	−0.50 (0.1440)	0.25 (0.4945)	−0.41 (0.2402)	0.09 (0.8153)	0.07 (0.8456)	−0.45 (0.1977)	−0.49 (0.1494)	−0.47 (0.1669)
LAScd_ED (%)	0.25 (0.4936)	0.34 (0.3342)	−0.26 (0.4758)	0.06 (0.8633)	0.04 (0.9065)	−0.10 (0.7842)	0.50 (0.1369)	0.36 (0.3070)	0.58 (0.0759)
LASct_ED (%)	0.39 (0.2718)	0.26 (0.4709)	−0.03 (0.9429)	0.42 (0.2236)	−0.13 (0.7277)	0.001 (0.9987)	0.05 (0.9004)	0.24 (0.5081)	0.03 (0.9337)
LASr_AC (%)	−0.48 (0.1528)	−0.49 (0.1476)	0.29 (0.4208)	−0.35 (0.3274)	0.10 (0.7860)	0.08 (0.8345)	−0.50 (0.1390)	−0.50 (0.1390)	−0.51 (0.1279)
LAScd_AC (%)	0.14 (0.7022)	0.26 (0.4627)	−0.27 (0.4479)	−0.05 (0.8958)	0.02 (0.9532)	−0.09 (0.8039)	0.45 (0.1921)	0.29 (0.4167)	0.50 (0.1371)
LASct_AC (%)	0.37 (0.2872)	0.24 (0.5117)	0.01 (0.9773)	0.43 (0.2124)	−0.12 (0.7410)	0.02 (0.9613)	0.04 (0.9197)	0.22 (0.5484)	0.01 (0.9738)
RA (right atrium) strain metrics									
RASr_ED (%)	−0.28 (0.4390)	−0.42 (0.2220)	0.59 (0.0698)	−0.01 (0.9778)	0.49 (0.1482)	0.31 (0.3857)	−0.18 (0.6160)	−0.58 (0.0770)	−0.17 (0.6330)
RAScd_ED (%)	0.07 (0.8380)	0.27 (0.4523)	−0.51 (0.1288)	−0.23 (0.5305)	−0.07 (0.8403)	−0.59 (0.0737)	0.07 (0.8481)	0.45 (0.1962)	0.26 (0.4734)
RASct_ED (%)	0.30 (0.3936)	0.35 (0.3286)	−0.43 (0.2156)	0.19 (0.6045)	−0.72 (0.0178 *)	0.07 (0.8487)	0.18 (0.6216)	0.43 (0.2137)	−0.05 (0.8819)
RASr_AC (%)	−0.22 (0.5389)	−0.40 (0.2495)	0.57 (0.0856)	0.08 (0.8262)	0.32 (0.3662)	0.42 (0.2295)	−0.21 (0.5617)	−0.57 (0.0841)	−0.30 (0.3991)
RAScd_AC (%)	0.04 (0.9116)	0.24 (0.5096)	−0.50 (0.1412)	−0.23 (0.5305)	−0.01 (0.9738)	−0.58 (0.0790)	0.07 (0.8381)	0.41 (0.2425)	0.25 (0.4810)
RASct_AC (%)	0.34 (0.3387)	0.41 (0.2387)	−0.46 (0.1832)	0.17 (0.6330)	−0.64 (0.0445 *)	−0.001 (0.9970)	0.23 (0.5162)	0.50 (0.1403)	0.10 (0.7926)

RV: right ventricle; RV_EDV_3D (mL): right ventricle end-diastolic volume measured by 3D echocardiography; RV_ESV_3D (mL): right ventricle end-systolic volume measured by 3D echocardiography; RV_EF_3D (%): right ventricle ejection fraction measured by 3D echocardiography; RV_SV_3D (mL): right ventricle stroke volume measured by 3D echocardiography; RV_TAPSE_3D (mm): tricuspid annular plane systolic excursion obtained by 3D echocardiography; RV_FAC_3D (%): right ventricle fractional area change obtained by 3D echocardiography; RV_Dd_base_3D (mm): right ventricle basal diameter measured at the basal third of the right ventricle in end-diastole by 3D echocardiography; RV_Dd_mid_3D (mm): right ventricle mid-cavity diameter measured at the mid-ventricular level of the right ventricle in end-diastole by 3D echocardiography; RV_Ld_3D (mm): right ventricle longitudinal distance from the tricuspid annulus to the right ventricle apex in end-diastole measured by 3D echocardiography; LASr_ED (%): left atrial reservoir strain, end-diastole-referenced; LAScd_ED (%): left atrial conduit strain, end-diastole-referenced; LASct_ED (%): left atrial contraction strain, end-diastole-referenced; LASr_AC (%): left atrial reservoir strain, atrial contraction-referenced; LAScd_AC (%): left atrial conduit strain, atrial contraction-referenced; LASct_AC (%): left atrial contraction strain, atrial contraction-referenced; RASr_ED (%): right atrial reservoir strain, end-diastole-referenced; RAScd_ED (%): right atrial conduit strain, end-diastole-referenced; RASct_ED (%): right atrial contraction strain, end-diastole-referenced; RASr_AC (%): right atrial reservoir strain, atrial contraction-referenced: RAScd_AC (%): right atrial conduit strain, atrial contraction-referenced; RASct_AC (%): right atrial contraction strain, atrial contraction-referenced; * significant result, *p* < 0.05.

## Data Availability

The original contributions presented in this study are included in the article. Further inquiries can be directed to the corresponding authors.

## Supplementary Materials

The following supporting information can be downloaded at: https://www.mdpi.com/article/10.3390/jcm15218524/s1, Table S1: Distributions of hemodynamic measurements in the PAH-CHD group. Legend: Data are summarized as mean (SD) or median [quartile1, quartile 3]; PVRi (WU/m2): pulmonary vascular resistance index (Wood Units/meter square); PVRi NO (WU/m2): pulmonary vascular resistance index post nitric oxide administration; Rp/Rs: the ratio of pulmonary resistance to systemic resistance; mPAP (mmHg): mean pulmonary artery pressure measured by catheterization; mPAP NO (mmHg): mean pulmonary artery pressure post nitric oxide administration; PCWP (mmHg): pulmonary capillary wedge pressure; RAP (mmHg): right atrial pressure; Qp/Qs: the ratio of pulmonary blow flow to systemic blood flow. Table S2. Distributions of indexed RV volumetric parameters between control and PAH-CHD groups. Legend: Data were presented as mean (SD); SD: standard deviation; *p*-values were derived from Type-III ANCOVA models, adjusted for BSA z-score; * Significant result, *p* < 0.05. RV_EDV_3D (mL): right ventricle end-diastolic volume measured by 3D echocardiography indexed; RV_ESV_3D (mL): right ventricle end-systolic volume measured by 3D echocardiography indexed; RV_SV_3D (mL): right ventricle stroke volume measured by 3D echocardiography indexed.

## Abbreviations

The following abbreviations are used in this manuscript:

2D	Two-dimensional
3D	Three-dimensional
3DE	Three-dimensional echocardiography
6MWT	Six-minute walk test
A2C	Apical two-chamber view
A3C	Apical three-chamber view
A4C	Apical four-chamber view
AC	Atrial contraction
ARV	Apical right ventricle segment longitudinal strain
ASD	Atrial septal defect
AUC	Area under the curve
AVD	Atrioventricular septal defect
BMI	Body mass index
BSA	Body surface area
BRV	Basal right ventricle segment longitudinal strain
CHD	Congenital heart disease
dBP	Diastolic blood pressure
ED	End-diastole
FAC	Fractional area change
HR	Heart rate
LA	Left atrium
LAScd_AC	Left atrial conduit strain, atrial contraction-referenced
LAScd_ED	Left atrial conduit strain, end-diastole-referenced
LASct_AC	Left atrial contraction strain, atrial contraction-referenced
LASct_ED	Left atrial contraction strain, end-diastole-referenced
LASr_AC	Left atrial reservoir strain, atrial contraction-referenced
LASr_ED	Left atrial reservoir strain, end-diastole-referenced
LV	Left ventricle
LV_AAL	Left ventricle apical anterolateral segment longitudinal strain
LV_AIS	Left ventricle apical inferoseptal segment longitudinal strain
LV_ED_mass_3D	Three-dimensional left ventricular end-diastolic mass
LV_EDL_3D	Three-dimensional left ventricular end-diastolic length
LV_EDV_3D	Left ventricle end-diastolic volume measured by 3D echocardiography
LV_EF_3D	Left ventricle ejection fraction measured by 3D echocardiography
LV_ESL_3D	Three-dimensional left ventricular end-systolic length
LV_ESV_3D	Left ventricle end-systolic volume measured by 3D echocardiography
LV_GLS	Left ventriculat global longitudinal strain
LV_SV_3D	Three-dimensional left ventricular stroke volume
MAPSE	Mitral annular plane systolic excursion
Mi_A′	Mitral annular late diastolic velocity (A′ wave) obtained by tissue Doppler echocardiography
Mi_E/A	Ratio between early and late mitral inflow velocity
Mi_E/E′	Ratio between early mitral inflow velocity and mitral annular early diastolic velocity
Mi_E′	Mitral annular early diastolic velocity (E′ wave) obtained by tissue Doppler echocardiography
Mi_IVCT	Mitral isovolumic contraction time
Mi_IVRT	Mitral isovolumic relaxation time
Mi_S	Mitral annular systolic velocity (S wave) obtained by tissue Doppler echocardiography
MRV	Mid right ventricle segment longitudinal strain
PAH	Pulmonary arterial hypertension
PAH-CHD	Pulmonary arterial hypertension associated with congenital heart disease
PDA	Patent ductus arteriosus
RA	Right atrium
RAScd_AC	Right atrial conduit strain, atrial contraction-referenced
RAScd_ED	Right atrial conduit strain, end-diastole-referenced
RASct_AC	Right atrial contraction strain, atrial contraction-referenced
RASct_ED	Right atrial contraction strain, end-diastole-referenced
RASr_AC	Right atrial reservoir strain, atrial contraction-referenced
RASr_ED	Right atrial reservoir strain, end-diastole-referenced
ROC	Receiver Operating Characteristic
RV	Right ventricle
RV4CSL	Right ventricle apical four-chamber view total strain
RVFWSL	Right ventricular free wall longitudinal strain
RV_Dd_base_3D	Right ventricle basal diameter measured at the basal third of the right ventricle in end-diastole by 3D echocardiography
RV_Dd_mid_3D	Right ventricle mid-cavity diameter measured at the mid-ventricular level of the right ventricle in end-diastole by 3D echocardiography
RV_EDV_3D	Right ventricle end-diastolic volume measured by 3D echocardiography
RV_EF_3D	Right ventricle ejection fraction measured by 3D echocardiography
RV_ESV_3D	Right ventricle end-systolic volume measured by 3D echocardiography
RV_FAC_2D	Right ventricle fractional area change obtained by 2D echocardiography
RV_FAC_3D	Right ventricle fractional area change obtained by 3D echocardiography
RV_Ld_3D	Right ventricle longitudinal distance from the tricuspid annulus to the right ventricle apex in end-diastole measured by 3D echocardiography
RV_SV_3D	Right ventricle stroke volume measured by 3D echocardiography
RV_TAPSE_3D	Tricuspid annular plane systolic excursion obtained by 3D echocardiography
RV_Tei_2D	Right ventricular myocardial performance index obtained by 2D echocardiography
SaO_2_	Peripheral oxygen saturation
sBP	Systolic blood pressure
Scd	Conduit strain
Sct	Contraction strain
SD	Standard deviation
Sr	Reservoir strain
STE	Speckle-tracking echocardiography
SV	Stroke volume
TAPSE	Tricuspid annular plane systolic excursion
TAPSE_2D	Tricuspid annular plane systolic excursion obtained by 2D echocardiography
Tei	Myocardial performance index
TGA	Transposition of the great arteries
Tr_A′	Tricuspid annular latediastolic velocity (A′ wave) obtained by tissue Doppler echocardiography
Tr_E/A	Ratio between early and late tricuspid inflow velocity
Tr_E/E′	Ratio between early tricuspid inflow velocity and tricuspid annular early diastolic velocity (E/E′)
Tr_E′	Tricuspid annular early diastolic velocity (E′ wave) obtained by tissue Doppler echocardiography
Tr_IVCT	Tricuspid isovolumic contraction time
Tr_IVRT	Tricuspid isovolumic relaxation time
Tr_S	Tricuspid annular systolic velocity (S wave) obtained by tissue Doppler echocardiography
VSD	Ventricular septal defect
WHO-FC	World Health Organization functional class
