# Supplementary material for: Right Ventricular Remodeling Using Three-Dimensional Echocardiography in Pediatric Pulmonary Arterial Hypertension Associated with Congenital Heart Disease: A Case–Control Study
Source: J Clin Med. 2026 Nov 2;15(21):8524. doi: 10.3390/jcm15218524 (PMC13764495; doi:10.3390/jcm15218524)
Supplement: Supplementary file 1 [file jcm-15-08524-s001.zip › jcm-4636939-supplementary.pdf]

Table S1. Distributions of hemodynamic measurements in the PAH-CHD group

	**PAH-CHD group ($n_2 = 11$)**
PVRi (WU/m2)	12.06 (4.06)
PVRi NO (WU)	10.36 (5.77)
Rp/Rs	1.04 [0.49, 1.42]
mPAP (mmHg)	48.09 (8.15)
mPAP NO (mmHg)	48.45 (11.81)
PCWP (mmHg)	5.18 (1.89)
RAP (mmHg)	8 (1.48)
Qp/Qs	0.75 (0.35)

Data are summarized as mean (SD) or median [quartile1, quartile 3];; PVRi (WU/m2): pulmonary vascular resistance index (Wood Units/meter square); PVRi NO (WU/m2): pulmonary vascular resistance index post nitric oxide administration; Rp/Rs: the ratio of pulmonary resistance to systemic resistance ; mPAP (mmHg): mean pulmonary artery pressure measured by catheterization; mPAP NO (mmHg): mean pulmonary artery pressure post nitric oxide administration; PCWP (mmHg): pulmonary capillary wedge pressure; RAP (mmHg): right atrial pressure; Qp/Qs: the ratio of pulmonary blow flow to systemic blood flow

Table S2. Distributions of indexed RV volumetric parameters between control and PAH-CHD groups

	**Control group ($n_1 = 50$)**	**PAH-CHD group ($n_2 = 11$)**	**p-value**
RV_EDVi_3D (mL/m^2)	65.12 (13.61)	95.03 (18.43)	0.0001*
RV_ESVi_3D (mL/m^2)	29.02 (8.17)	50.10 (10.84)	<0.0001*
RV_SVi_3D (mL/m^2)	36.17 (7.47)	48.12 (10.26)	0.0163*

*Data were presented as mean (SD); SD: standard deviation; p-values were derived from Student-t test for independent samples; * Significant result, $p < 0.05$.*
